# A Practical Guide to Using Biologics in Pediatric Dermatology

**DOI:** 10.1177/12034754231222415

**Published:** 2024-01-16

**Authors:** Annie George, Rafael Paolo Lansang, Perla Lansang, Melinda Gooderham

**Affiliations:** 1 Temerty School of Medicine, University of Toronto, Toronto, ON, Canada; 2 Michael G. Degroote School of Medicine, McMaster University, Hamilton, ON, Canada; 3 Division of Dermatology, Sunnybrook Health Sciences Centre, University of Toronto, Toronto, ON, Canada; 4 SKiN Centre for Dermatology, Peterborough, ON, Canada; 5 Probity Medical Research, Waterloo, ON, Canada; 6 Department of Medicine, Queen’s University, Kingston, ON, Canada

**Keywords:** psoriasis, hidradenitis suppurativa, chronic spontaneous urticaria, atopic dermatitis, biologics, pediatrics

## Abstract

Psoriasis, hidradenitis suppurativa (HS), atopic dermatitis (AD), and chronic spontaneous urticaria (CSU), are common, immune-mediated, chronic, inflammatory skin diseases that can affect the pediatric population. While there is adequate evidence supporting the use of biologics in pediatric patients, concerns regarding safety and efficacy amongst healthcare providers are not uncommon. However, new emerging evidence in this population highlights the safety of biologic therapy, making it crucial to review and establish a practical guide for their use. This article describes a methodological framework for initiating biologics in the management of pediatric psoriasis, HS, AD, and CSU, with a special focus on baseline work-up, monitoring, dosing, and considerations in this population.

## Introduction

Pediatric skin conditions, such as psoriasis, hidradenitis suppurativa (HS), atopic dermatitis (AD), and chronic spontaneous urticaria (CSU), have a significant impact on patient quality of life and development.^1,2^ Chronic skin diseases are associated with mood disorders in children and adults, including anxiety/depression.^3,4^ Furthermore, pediatric patients with chronic diseases are at increased risk for strained family relations, poor self-image, psychiatric comorbidities, stigmatization, and eventual suicidal behaviour.^5^ These conditions therefore warrant prompt and effective treatment to reduce physical and psychological comorbidities. Classic, non-targeted treatments such as topical therapies, conventional systemic non-biologic therapies, or phototherapy have historically been the first line of treatment for pediatric patients. Although these traditional agents have been in use for many years, inadequate response and intolerance are not uncommon.^6^

Certain biologic therapies previously approved for adults with psoriasis, HS, AD, and CSU are now also approved for the management of the pediatric population. Although there may be a hesitancy amongst some providers to initiate biologics in pediatric patients, results from recent studies have proven both the efficacy and favourable safety profile of biologic therapies in the pediatric population.^7^ Furthermore, new emerging evidence suggests that biologic therapy could potentially be safer than conventional systemic therapies.^8^ These advancements in treatment and availability of new therapies make it crucial to review and update previously established treatment guidelines in pediatric patients.^9^ This review updates prior guidelines on pediatric psoriasis management^9^ and extends its scope to include newly available biologics for this age group, while also considering application of biologics in other pediatric inflammatory skin conditions. This review serves to provide a practical guide for the clinician initiating biologics in the management of psoriasis, HS, AD, and CSU, with a special focus on baseline work-up, monitoring, dosing, and considerations for currently approved biologics in the pediatric population.

## Psoriasis

Psoriasis is a chronic, immune-mediated, inflammatory dermatosis characterized by the development of well-circumscribed, erythematous, scaly plaques. Psoriasis affects 2% to 5% of the general population, with up to one-third of the cases beginning in childhood.^9^ Although topical therapies, phototherapy, cyclosporine, methotrexate, and acitretin have been the mainstay of treatment for moderate-to-severe pediatric psoriasis, 4 biologic therapies (etanercept, ixekizumab, secukinumab, ustekinumab) have received a pediatric indication by the US Food and Drug Administration (FDA) and Health Canada as early as 2015 for ustekinumab and 2017 for etanercept.^10^ Table 1 summarizes the attributes of the 4 Health Canada-approved biologics for pediatric psoriasis. Of note, adalimumab is also approved for use in pediatric psoriasis (ages 4 years and above) in Europe since 2015 but does not have this indication in Canada.^11^

**Table 1.** Systemic Biologics for Pediatric Psoriasis: Target, Indication, Dosing, Clinical Measures, Patient Reported Outcomes, and Laboratory Measures.^12-15^

Therapy (year of pediatric approval)*	Target	Indication	Dosing and frequency	Clinical measures	Patient reported outcomes	Laboratory measures
Etanercept (2017)^*^	TNFα	Psoriasis (4–17 years)	0.8 mg/kg (max 50 mg/dose) every week**	PASI, IGA, BSA, weight	CDLQI	CBC, renal (Cr, BUN), liver (ALT, AST, ALP, GGT, bilirubin), TB, serologic studies (HBV, HCV, VZV titres^#^), vaccine status
Ixekizumab (2021)^*^	IL-17A	Psoriasis (6–17 years)	**<25** **kg:** 40 mg at week 0, 20 mg every 4 weeks **25-50** **kg:** 80 mg at week 0, 40 mg every 4 weeks **≥50** **kg:** 160 mg at week 0, 80 mg every 4 weeks	PASI, IGA, BSA, weight	CDLQI	CBC, renal (Cr, BUN), liver (ALT, AST, ALP, GGT, bilirubin), TB, serologic studies (HBV, HCV, VZV titres^#^), vaccine status
Secukinumab (2022)^*^	IL-17A	Psoriasis (6–17 years)	**<50 kg:** 75 mg **≥50 kg:** 150 mg at weeks 0, 1, 2, 3, and 4, and every 4 weeks thereafter	PASI, IGA, BSA, weight	CDLQI	CBC, renal (Cr, BUN), liver (ALT, AST, ALP, GGT, bilirubin), TB, serologic studies (HBV, HCV, VZV titres^#^), vaccine status
Ustekinumab (2015, ages 12-17 years; 2020, ages 6-11 years)^*^	IL-12/23	Psoriasis (6–17 years)	**<60** **kg:** 0.75 mg/kg **60-100** **kg:** 45 mg **≥100** **kg:** 90 mg at weeks 0, 4, and every 12 weeks thereafter	PASI, IGA, BSA, weight	CDLQI	CBC, renal (Cr, BUN), liver (ALT, AST, ALP, GGT, bilirubin), TB, serologic studies (HBV, HCV, VZV titres^#^), vaccine status

Abbreviations: ALP, alkaline phosphatase; ALT, alanine transaminase; AST, aspartate aminotransferase; BSA, body surface area; BUN, blood urea nitrogen; CBC, complete blood count; CDLQI, Children’s Dermatology Life Quality Index; Cr, creatinine; GGT, gamma-glutamyl transferase; HBV, hepatitis B virus; HCV, hepatitis C virus; IGA, Investigators Global Assessment; IL, interleukin; PASI, Psoriasis Area and Severity Index; TB, tuberculosis; VZV, varicella-zoster virus.

* Notice of compliance dates available from: https://health-products.canada.ca/noc-ac/, accessed August 12, 2023.

** SureClick^®^ autoinjector can be used if **≥**63 kg.

# VZV titres should only be ordered when vaccination status is unknown.

Etanercept, a TNF receptor fusion protein, has been used effectively for several years in the management of juvenile idiopathic arthritis and is approved for pediatric psoriasis in patients aged 4 to 17 years. A phase III trial showed 57% of patients (4 to 17 years of age) receiving etanercept at 0.8 mg/kg (to a maximum of 50 mg) achieved 75% improvement in the Psoriasis Area and Severity Index (PASI 75) and 27% achieved 90% improvement (PASI 90), which was significantly higher compared to patients receiving placebo.^16^ The most common side effects of etanercept in the pediatric study included mild and transient injection site reactions, an increased risk of upper respiratory tract infections (URTI), headache, and nasopharyngitis. There were no serious adverse events (SAEs) in the placebo-controlled period, and few infectious SAEs were noted in the open-label period.^16^ The adverse event profile in the pediatric population was similar to that reported in the adult population.^17^

A phase III trial of ixekizumab, a monoclonal antibody targeting interleukin (IL)-17A, was found to be superior to placebo at 12 weeks of treatment in patients with psoriasis aged 6 to 17 years.^18^ Furthermore, a randomized controlled trial (RCT) of 139 pediatric patients with plaque psoriasis who completed ixekizumab (<25 kg: 40 mg at week 0, 20 mg every 4 weeks; 25-50 kg: 80 mg at week 0, 40 mg every 4 weeks; ≥50 kg: 160 mg at week 0, 80 mg every 4 weeks) showed that improvements were sustained through week 108, with patients achieving PASI 75 (91.7%), PASI 90 (79.0%), PASI 100 (55.1%), static Physician’s Global Assessment (sPGA) 0 or 1 (78.3%), and sPGA 0 (52.4%).^19^ This trial reported the safety profile of ixekizumab to be consistent with previously reported data, with common side effects including injection site reactions (20%), and infections. Inflammatory bowel disease (IBD) was reported in 2% of the all-ixekizumab exposed group (adjudicated as Crohn’s disease) but no cases of candidiasis were reported during the study.^19^

Secukinumab, another monoclonal antibody targeting IL-17A, has proven sustained efficacy with a favourable safety profile in pediatric patients with moderate-to-severe psoriasis. A phase III study has shown secukinumab (75 mg for patients <50 kg, 150 mg for patients ≥50 kg) to be superior compared to placebo in pediatric patients (6–17 years of age). The study found 80% of the patients receiving secukinumab reached PASI 75 compared to 14.6% of patients receiving placebo at week 12, with the efficacy sustained to week 52. Furthermore, secukinumab was consistent in terms of safety profile with the adult phase III studies—the most commonly reported side effects included pharyngitis, rhinitis, nasopharyngitis, and URTI. There were no cases of IBD reported and rates of candidiasis were low at 1.8%.^20^

The monoclonal antibody ustekinumab targets the p40 subunit common to IL-12 and IL-23, which are key regulatory cytokines significant in the pathogenesis of psoriasis. In the phase III CADMUS Junior study, ustekinumab (<60 kg: 0.75 mg/kg; ≥60 to ≤100 kg: 45 mg; >100 kg: 90 mg) was proven to be effective in pediatric patients (6-12 years of age) with moderate-to-severe psoriasis with 77% of patients achieving an Investigators Global Assessment of clear/almost clear (IGA 0/1) response at week 12.^21^ Furthermore, the phase III CADMUS trial showed 69.4% of patients (12–17 years of age) receiving ustekinumab (≤60 kg: 0.75 mg/kg; <60 to ≤100 kg: 45 mg; >100 kg: 90 mg) achieved PGA 0/1 versus 5.4% for placebo.^22^ Commonly reported side effects of ustekinumab in the pediatric population included transient injection site erythema, nasopharyngitis, pharyngitis, and URTI.^21^

## Hidradenitis Suppurativa

HS is a chronic skin disease of the hair follicle with a prevalence of 14 to 20 per 100,000 in the pediatric population.^23^ Adalimumab, a monoclonal antibody targeting TNFα, has been approved for the management of HS in pediatric patients, although RCTs evaluating its efficacy and safety were initially performed in the adult population and have not been performed in the pediatric population.^24^ A systematic review evaluating the efficacy of biologics in pediatric patients with HS reported adalimumab was the most commonly used biologic in this population. Furthermore, 18% of pediatric patients who received adalimumab experienced complete resolution, and 53% achieved partial resolution.^25^ Additionally, adalimumab has a 16-year-long safety profile in various pediatric diseases. Commonly reported side effects include injection site reactions, headache, URTI and myalgia, and have a similar profile to the adult studies.^24,26^ The treatment indication, dosing, and frequency for managing HS in pediatric patients is included in Table 2.

**Table 2.** Systemic Biologics: Indications, Dosing, Clinical Measures, Patient Reported Outcomes, and Laboratory Measures.^27,28-30^

Therapy (year of pediatric approval)*	Indication	Dosing and frequency	Clinical measures	Patient reported outcomes	Laboratory measures
Adalimumab (2018)	HS (≥12 years, ≥30 kg)	**30 to <60** **kg:** Day 1: 80 mg, Day 8: 40 mg every 2 weeks ≥60 kg: Day 1: 160 mg, Day 15: 80 mg, Day 29: 40 mg weekly	HiSCR (AN count) weight	CDLQI	CBC, renal (Cr, BUN), liver (ALT, AST, ALP, GGT, bilirubin), TB, serologic studies (HBV, HCV, VZV titres^#^), vaccine status
Dupilumab (2019, 12-17 years; 2021, 6-11 years; 2023, 6 months-5 years)	AD (6 months-17 years)	**5 to <15** **kg:** Day 1: 200 mg, then 200 mg every 4 weeks 15 to <30 kg: Day 1: 300 mg (6 months-5 years) or 600 mg (6-17 years), then 300 mg every 4 weeks 30 to <60 kg: Day 1: 400 mg, then 200 mg every 2 weeks ≥60 kg: Day 1: 600 mg, then 300 mg every 2 weeks	EASI, IGA, SCORAD weight	POEM, sleep NRS, itch NRS, CDLQI	Not required except vaccine status
Tralokinumab (2023)	AD (12-17 years)	Day 1: 600 mg, then 300 mg every 2 weeks *Note*: 300 mg every 4 weeks can be considered in patients who achieve IGA 0/1	EASI, IGA, SCORAD	POEM, sleep NRS, itch NRS, CDLQI	Not required except vaccine status
Omalizumab (2014)	CSU (≥12 years)	150-300 mg SC, then every 4 weeks	UAS7	CDLQI	Not required except vaccine status

Abbreviations: AD, atopic dermatitis; ALP, alkaline phosphatase; ALT, alanine transaminase; AN, abscess nodule count; AST, aspartate aminotransferase; BSA, body surface area; BUN, blood urea nitrogen; CBC, complete blood count; CDLQI, Children’s Dermatology Life Quality Index; Cr, creatinine; CSU, chronic spontaneous urticaria; EASI, Eczema Area and Severity Index; GGT, gamma-glutamyl transferase; HBV, hepatitis B virus; HCV, hepatitis C virus; HiSCR, Hidradenitis Suppurativa Clinical Response; HS, hidradenitis suppurativa; IGA, Investigator Global Assessment; NRS, numeric rating scale; PASI, Psoriasis Area and Severity Index; POEM, Patient Oriented Eczema Measure; SCORAD, Scoring Atopic Dermatitis; TB, tuberculosis; UAS7, Urticaria Activity Score; VZV, varicella-zoster virus.

* Notice of compliance dates available from: https://health-products.canada.ca/noc-ac/, accessed August 12, 2023.

# VZV titres should only be ordered when vaccination status is unknown.

## Atopic Dermatitis

AD is a chronic systemic inflammatory condition affecting the skin with a childhood prevalence of at least 10% in North America.^31^ Considering the recurrent nature of this systemic inflammatory disease, active therapies to manage flares and maintenance therapies to prevent future flares and promote integrity of the skin barrier are required. Although the treatment for AD has typically been with topical therapies and off-label systemic therapies, treatment with biologics has become more common. Dupilumab, a monoclonal antibody which targets the IL-4R alpha subunit, has been approved for AD treatment for pediatric patients over 6 months of age.^32^ Dupilumab inhibits IL-4 and IL-13, which are key cytokines in the pathogenesis of AD. Dupilumab was found to have a favourable efficacy and safety profile in the pediatric and adolescent population (Table 2). The phase III program (including LIBERTY AD PRESCHOOL, LIBERTY AD-PEDS, and LIBERTY AD-ADOL) showed significant improvement with 75% improvement in the Eczema Area and Severity Index from baseline (EASI-75) and IGA 0/1 amongst pediatric and adolescent patients treated with dupilumab compared to placebo.^33-35^ It is important to note that conjunctivitis and injection site reactions were reported more commonly in pediatric patients with AD treated with dupilumab compared to the placebo group, but rates of non-herpetic skin infections were lower in the dupilumab treated group.^35,36^

Tralokinumab, a monoclonal antibody to IL-13, is also now approved in adolescents aged 12 to 17 years in Canada and Europe.^37^ In the phase III trial, ECZTRA 6, tralokinumab at the 150 and 300 mg dose were both more effective than placebo at week 16, with more patients on tralokinumab achieving an IGA score 0/1, EASI-75, and Adolescent Worst Pruritus Numeric Rating Scale (NRS) reduction of 4 points or more from baseline.^38^ Dosing for adolescents is the same as dosing recommended for adults, with a loading dose of 600 mg on Day 1 followed by 300 mg every other week. For patients who achieve IGA 0/1 by week 16, a reduced dosing frequency can be considered (Table 2).^37^ The most common side effects in ECZTRA 6 included URTI, AD exacerbation, injection site reaction, and headache. Rates of conjunctivitis were low, 3% to 4% versus 2%, in the tralokinumab group compared to the placebo group, respectively.^38^

## Chronic Spontaneous Urticaria

CSU is characterized by the recurrence of transient angioedema and/or wheals persisting for at least 6 weeks, with a prevalence of 0.1% to 0.3% in the pediatric population.^39^ According to the updated EAACI/GA^2^LEN/EuroGuiDerm/APAAACI international guidelines published in 2022, initial steps for management include avoiding identifiable triggers and physical stimuli, followed by pharmacological therapies such as non-sedating second-generation H1-antihistamines.^40^ The guidelines recommend with strong consensus to using the same treatment algorithm with caution (eg, weight-adjusted dosage) in children with chronic urticaria. In 2014, omalizumab was approved by Health Canada and the FDA for the treatment of resistant CSU in patients ≥12-year-old, with a standard dosage of 150 to 300 mg subcutaneously every 4 weeks (Table 2).^40-42^ In 3 double-blind phase III RCTs, results show that omalizumab 300 mg injections were effective in reducing CSU symptoms compared to placebo in patients ≥12 years of age, with a range of 51.9% to 66% achieving an Urticaria Activity Score (UAS7) ≤6 at week 12 (*P* < .001).^43-46^ Studies, including those with children under the age of 12, have shown omalizumab to have a favourable safety profile in pediatric patients.^41^ The most commonly reported side effects in the adolescents and adult CSU studies include injection site reactions, URTI, viral infections, sinusitis, and headache.^43^

## Baseline Work-Up and Monitoring for Systemic Biologics

Disease severity and treatment response can be assessed effectively using standardized clinical measures and patient reported outcomes specific to the disease. Pediatric patients should be assessed for disease severity using tools such as body surface area (BSA), PGA or IGA, PASI, EASI, Scoring Atopic Dermatitis (SCORAD), Hidradenitis Suppurativa Clinical Response (HiSCR), and UAS7.^46-48^ Furthermore, the patient’s emotional, social, and school functioning, as well as their satisfaction with treatment can be explored using patient reported outcomes such as the Children’s Dermatology Life Quality Index in all these inflammatory diseases.^5^ Patient-Oriented Eczema Measure (POEM), NRS-itch, and NRS-sleep are additional standardized tools used to assess patient-reported symptoms amongst pediatric patients with AD.^49^ The clinical assessment and patient-reported tools are outlined in Table 3 based on disease type.

**Table 3.** Baseline Work-Up for Biologic Therapies in Pediatric Patients.

	Psoriasis	Hidradenitis suppurativa	Atopic dermatitis	Chronic spontaneous urticaria
Clinical measures	PASI, IGA, BSA, weight	HiSCR (AN count), weight	EASI, IGA, SCORAD, weight	UAS7
Patient-reported outcomes	CDLQI	CDLQI	POEM, sleep NRS, itch NRS, CDLQI	CDLQI
Laboratory measures	CBC, renal (Cr, BUN), liver (ALT, AST, ALP, GGT, bilirubin), TB, serologic studies (HBV, HCV, VZV titres^#^), vaccine status	CBC, renal (Cr, BUN), liver (ALT, AST, ALP, GGT, bilirubin), TB, serologic studies (HBV, HCV, VZV titres), vaccine status	Not required except vaccine status	Not required except vaccine status

Abbreviations: ALP, Alkaline phosphatase; ALT, alanine transaminase; AN, abscess nodule count; AST, aspartate aminotransferase; BSA, body surface area; BUN, blood urea nitrogen; CBC, complete blood count; CDLQI, Children’s Dermatology Life Quality Index; Cr, creatinine; CSU, chronic spontaneous urticaria; EASI, Eczema Area and Severity Index; GGT, gamma-glutamyl transferase; HBV, hepatitis B virus; HCV, hepatitis C virus; HiSCR, Hidradenitis Suppurativa Clinical Response; HS, hidradenitis suppurativa; IGA, Investigator Global Assessment; NRS, numeric rating scale; PASI, Psoriasis Area and Severity Index; POEM, Patient-Oriented Eczema Measure; SCORAD, Scoring Atopic Dermatitis; TB, tuberculosis; UAS7, Urticaria Activity Score; VZV, varicella-zoster virus.

# VZV titres should only be ordered when vaccination status is unknown.

Prior to the initiation of biologics amongst patients with psoriasis and HS, patients should undergo baseline blood work, as well as screening for infections such as hepatitis and tuberculosis (TB) (Table 3). In the case of a positive TB test, it is recommended patients be started on prophylactic therapy or referred to an infectious disease specialist. It is important to note that no baseline laboratory measures are indicated in patients with AD or CSU, unless other systemic therapies are being considered for management purposes. In terms of routine monitoring while on therapy, laboratory measures are not indicated for patients with psoriasis, HS, AD, or CSU; however, repeated TB tests may be required for high-risk patients on a case-by-case basis (eg, exposure to persons with TB, travel).

According to the National Advisory Committee on Immunization guidelines, children should be up-to-date on their immunizations prior to starting biologic therapy (Table 4).^50^ In patients whose parents choose not to vaccinate, caution should be exercised. In the case of unknown varicella immunization status, measuring varicella zoster titres can be considered. If negative, vaccination can be recommended. Live vaccines are not recommended while receiving biological therapies because biologics may reduce the immune response, potentially leading to inadequate vaccine effectiveness and an increased risk of infections. This precaution is taken to ensure patient safety and proper vaccination outcomes. In the case where live vaccines are required, it is recommended the biologic therapy be stopped for at least 3 months before administering the vaccine. Biologics can then be reintroduced to the treatment regimen 2 to 4 weeks post vaccine administration. However, this decision should be made on a case-by-case basis as benefits of remaining on therapy may outweigh the risks in certain situations. It is recommended that patients on biologic therapy receive appropriate non-live vaccines including annual influenza and COVID-19 boosters as per guidelines.^50^

**Table 4.** Canadian Routine Childhood Immunization Schedule, Infants, and Children (Birth to 17 Years of Age) From the National Advisory Committee on Immunization.

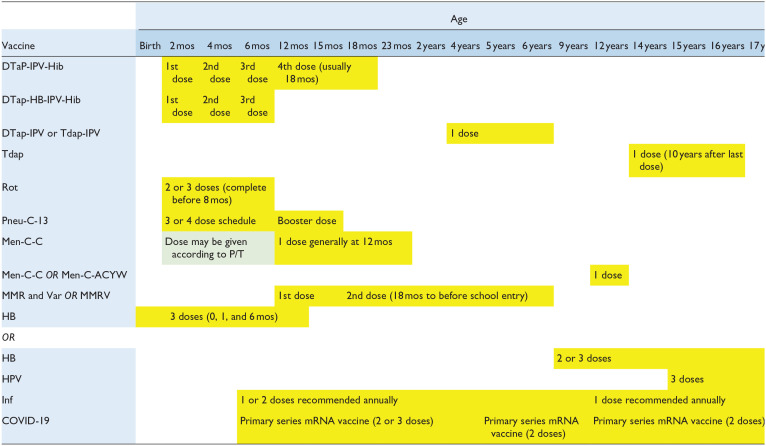

Abbreviations: DTap-HB-IPV-Hib, diphtheria toxoid–tetanus toxoid–acellular pertussis–hepatitis B–inactivated polio–Haemophilus influenzae type b; DTap-IPV, diphtheria toxoid–tetanus toxoid–acellular pertussis–inactivated polio; DTaP-IPV-Hib, diphtheria toxoid–tetanus toxoid–acellular pertussis–inactivated polio–Haemophilus influenzae type b; HB, hepatitis B; HPV, human papillomavirus; Inf, influenza; Men-C-ACYW, meningococcal conjugate quadrivalent; Men-C-C, meningococcal conjugate monovalent; MMR, measles–mumps–rubella; MMRV, measles–mumps–rubella–varicella; mos, months; Pneu-C-13, pneumococcal conjugate 13-valent; P/T, province or territory; Rot, rotavirus; Tdap, tetanus toxoid–reduced diphtheria toxoid–reduced acellular pertussis; Tdap-IPV, tetanus toxoid–reduced diphtheria toxoid–reduced acellular pertussis–inactivated polio.

## Practical Considerations

Prescribing biologic medications for children with inflammatory skin diseases requires careful consideration of various practical aspects. When it comes to injections, it is essential to provide caregivers guidance on proper administration techniques to ensure optimal drug delivery.^51^ Clinicians can offer tips to minimize injection discomfort, such as ensuring drug is at ambient temperature before injection, rotation of injection sites, using a thinner needle when possible, or applying ice or topical anaesthetics prior to injection. Additionally, educating patients and their caregivers on the management of potential injection site reactions, including pruritus, redness, or swelling, can help improve adherence and minimize treatment disruptions. Pre-treatment of injection sites with topical steroids or taking oral antihistamines prior to drug administration may be considered in some patients.^52^

In surgical scenarios, it is crucial to maintain the biologic treatment schedule whenever possible, particularly for medications like dupilumab, tralokinumab, or omalizumab. However, for biologics like anti-TNF, anti-12/23, and anti-17 agents, which may increase the risk of infection, consultation with the surgeon is recommended, weighing the risks, and benefits of each clinical scenario. Furthermore, when dealing with infections or febrile illnesses, a cautious approach is paramount. Clinicians should advise holding all biologics during active infections. In cases of febrile illnesses with uncertain sources of infection, a careful risk-to-benefit assessment should be conducted, and biologic therapy may need to be temporarily paused until the infection is identified and appropriately treated to ensure the child’s well-being and optimal outcomes.

## Conclusion

Although health care providers may be hesitant toward initiating biologics earlier amongst pediatric patients with inflammatory skin conditions, multiple studies in multiple indications have proven biologic therapies to be effective and safe in this population. Here, we provide a practical guide for initiating currently approved biologics in pediatric patients with psoriasis, HS, AD, and CSU, for all biologics currently approved in Canada for pediatric indications; however, newer treatments are continuously being added to our arsenal of available therapies. This paper serves as a comprehensive guide for baseline work-up and monitoring, as well as immunization and practical considerations.
